# Optical photothermal infrared spectroscopy: A novel solution for rapid identification of antimicrobial resistance at the single-cell level *via* deuterium isotope labeling

**DOI:** 10.3389/fmicb.2023.1077106

**Published:** 2023-02-01

**Authors:** Sahand Shams, Cassio Lima, Yun Xu, Shwan Ahmed, Royston Goodacre, Howbeer Muhamadali

**Affiliations:** ^1^Centre for Metabolomics Research, Department of Biochemistry and Systems Biology, Institute of Systems, Molecular, and Integrative Biology, University of Liverpool, Liverpool, United Kingdom; ^2^Department of Environment and Quality Control, Kurdistan Institution for Strategic Studies and Scientific Research, Sulaymaniyah, Kurdistan Region, Iraq

**Keywords:** antimicrobial resistance, single-cell, microbiology, infrared spectroscopy, stable-isotope probing, Uropathogenic *Escherichia coli*

## Abstract

The rise and extensive spread of antimicrobial resistance (AMR) has become a growing concern, and a threat to the environment and human health globally. The majority of current AMR identification methods used in clinical setting are based on traditional microbiology culture-dependent techniques which are time-consuming or expensive to be implemented, thus appropriate antibiotic stewardship is provided retrospectively which means the first line of treatment is to hope that a broad-spectrum antibiotic works. Hence, culture-independent and single-cell technologies are needed to allow for rapid detection and identification of antimicrobial-resistant bacteria and to support a more targeted and effective antibiotic therapy preventing further development and spread of AMR. In this study, for the first time, a non-destructive phenotyping method of optical photothermal infrared (O-PTIR) spectroscopy, coupled with deuterium isotope probing (DIP) and multivariate statistical analysis was employed as a metabolic fingerprinting approach to detect AMR in Uropathogenic *Escherichia coli* (UPEC) at both single-cell and population levels. Principal component-discriminant function analysis (PC-DFA) of FT-IR and O-PTIR spectral data showed clear clustering patterns as a result of distinctive spectral shifts (C–D signature peaks) originating from deuterium incorporation into bacterial cells, allowing for rapid detection and classification of sensitive and resistant isolates at the single-cell level. Furthermore, the single-frequency images obtained using the C–D signature peak at 2,163 cm^−1^ clearly displayed the reduced ability of the trimethoprim-sensitive strain for incorporating deuterium when exposed to this antibiotic, compared to the untreated condition. Hence, the results of this study indicated that O-PTIR can be employed as an efficient tool for the rapid detection of AMR at the single-cell level.

## 1. Introduction

Since the 1980s, due to the extreme and indiscreet consumption of antimicrobial agents in both humans and animals, antimicrobial resistance (AMR) has emerged (Boerlin and Reid-Smith, 2008; Sengupta et al., 2013), and become a worldwide threat. If actions are not taken to address this issue, it is estimated that by 2050 more than 10 million people will die annually from AMR alone, which is more than diabetes or cancer with 1.5 million and 8.2 million annual death cases, respectively (O'Neill, 2016).

Along with the increase in the number of resistant pathogens, AMR detection methodologies have also advanced (Charteris et al., 2001), and for clinical application, the development of quick antibiotic susceptibility testing (AST) has become a priority (Kerremans et al., 2008; van Belkum et al., 2019). If AST is realized, then judicious antimicrobials can be used, which also opens up the possibility of using old antibiotics for therapy and thus reserving new antibiotics for emergency cases. However, generally, manual AST approaches are exceedingly time-consuming and overshadowed by the level of expertise and errors of labor during the sample analysis (Reller et al., 2009). Thus, to carry out AST of pathogens against multiple antimicrobial agents at the same time, automated AST systems are largely being used to reduce labor and time requirements (Shanmugakani et al., 2020). The approved automated AST systems use various antimicrobial panels and technologies designed for testing the antimicrobial susceptibility of Gram-positive as well as Gram-negative pathogens (Reller et al., 2009). This is while, all commercially available instruments require up to 24 h to provide AST results (Shanmugakani et al., 2020).

During the past two decades, there has been a significant improvement in molecular and genotyping approaches for AMR detection, which has revolutionized the diagnosis of bacterial infections (Templeton, 1992; Anjum et al., 2017). Accompanied by the development of polymerase chain reaction (PCR) and its application, several forms of nucleic acid amplification tests (NAATs) including isothermal amplification were designed for the identification of AMR to one or multiple types of antimicrobials (Deiman et al., 2008; Ao and Jenison, 2013; Rivoarilala et al., 2018; Xu et al., 2018). Additionally, although whole-genome sequencing allows for a comprehensive understanding of resistance to entirely recognized antimicrobials as well as mechanisms (Köser et al., 2014; Oniciuc et al., 2018), compared to phenotypic tests such as enzymatic activity-based tests, bacterial growth-based tests and immunoassays, it is more expensive, requires significant infrastructure and is difficult to implement and needs a certain level of proficiency (Shanmugakani et al., 2020).

The utilization of single-cell technologies may lead to the development of rapid AST systems and the reduction of turnaround time, especially as there is the tantalizing possibility of making measurements without the need to culture the pathogen. Moreover, primary recognition of AMR in the microbial cells will be valuable to guide antimicrobial therapeutic strategies and preventing the progression of infection in the healthcare system (Bhattacharya, 2013; Simões et al., 2016).

Fourier transform infrared (FT-IR) spectroscopy is one of the most popular techniques by which the biochemical profile of biological samples, based on the vibrations of various IR-active biomolecules such as proteins, nucleic acids, carbohydrates and lipids, can be studied (Baker et al., 2014). As a whole-organism fingerprinting method, FT-IR spectroscopy has thrived to assess the progress of antibiotic resistance in bacteria (Salman et al., 2017; Sharaha et al., 2017). Previous studies have also demonstrated the application of FT-IR spectroscopy for the detection and classification of bacterial cells at the population level (Muhamadali et al., 2016), the reader is directed to (AlMasoud et al., 2021) for a recent review on the applications of whole-organism fingerprinting methods. The coupling of an FT-IR spectrophotometer to an optical microscope, on the other hand, provided a system that is capable of doing FT-IR microspectroscopy, which is a powerful tool that allows for the analysis of samples at small scale (e.g. particles with a diameter less than 150 μm; Nicosia and Stoops, 2017). However, FT-IR microspectroscopy provides a spatial resolution of 7–14 μm and cannot distinguish structures smaller than a few micrometers due to the diffraction limit of the IR light, making it inefficient for imaging subcellular structures or single bacteria with dimensions typically ranging from 1 to 2 μm (Bechtel et al., 2014). To address these drawbacks, Optical Photothermal Infrared (O-PTIR) spectroscopy, which is a novel far-field infrared imaging technique that works on the basis of the photothermal effects of infrared radiation, was developed. O-PTIR provides chemical images at submicron spatial resolution and evaluates the photothermal response of a sample irradiated by an adjustable mid-IR laser beam (Lima et al., 2021) in such a way that a pulsed tunable quantum cascade laser (QCL) IR laser is guided onto the sample surface and then, the IR source is made collinear with a 532 nm detection laser (green). The collinear beams are focused on the sample surface through a microscope objective. When the IR is absorbed by the sample, the thermal response of the sample surface is consequently monitored by the green detection laser. The reflected green light returns to the detector while the IR signal is extracted (Kansiz et al., 2020).

Previous studies employed mid-infrared imaging techniques for bacterial investigation, such as the study by Lima et al., which used O-PTIR spectroscopy combined with stable-isotope probing (SIP), in particular ^13^C and ^15^N isotopes, for imaging isotopically labeled bacteria at single-cell level. Such an approach allows for monitoring the molecular vibrations affected by the incorporation of “heavy” atoms into cellular biochemical components (Lima et al., 2021). Xu et al. (2020) also reported the application of Raman-integrated optical mid-infrared photothermal microscopy for detecting biochemical changes of bacteria in response to erythromycin within 1 h of exposure.

SIP can be defined as the use of stable (nonradioactive) isotopes that can act as a tracer and be used to monitor different biochemical activities in various biological systems. These stable isotopes, which include ^2^H (deuterium), ^13^C, ^15^N and ^18^O, can be used during the growth phase of microorganisms so they can be incorporated into the structure of various biomolecules such as amino acids, fatty acids and nucleic acids (Muhamadali et al., 2015). In a previous study, we demonstrated the application of SIP, reverse SIP, and DIP combined with FT-IR and Raman spectroscopy, and secondary ion mass spectrometry (SIMS) for monitoring the metabolic activity of bacterial cells under various culturing conditions at the bulk level (Chisanga et al., 2021).

Previous studies have reported the use of deuterium oxide (D_2_O) combined with Raman microspectroscopy for the identification of active bacterial cells under specific growth conditions (Berry et al., 2015), to investigate cross feeding in microbial communities (Wang et al., 2016; Chisanga et al., 2021). Song et al. (2017) also reported the application of a similar strategy for the identification of bacteria resistant to carbenicillin and kanamycin, in samples isolated from the River Thames. However, to our best of knowledge so far, there are no studies in the literature reporting the combined application of DIP and O-PTIR spectroscopy for the detection of AMR at the single-cell level. Hence, in this study, for the first time, we have employed FT-IR and O-PTIR spectroscopy combined with DIP, and multivariate statistical analysis techniques for the detection of AMR in Uropathogenic *Escherichia coli* (UPEC) isolates at both population and single-cell levels.

## 2. Materials and methods

### 2.1. Chemicals, microorganisms, and growth conditions

All chemical compounds used in this study were purchased from Sigma-Aldrich (United Kingdom) unless otherwise indicated. In addition to *E. coli* MG1655 which was used as the standard susceptible strain to trimethoprim (negative control), different UPEC isolates were selected from Dawson et al. (2014) and grown on Luria-Bertani (LB) agar from −80°C glycerol stocks to obtain axenic colonies. Each isolate was subcultured 3 times on LB agar plates at 37°C overnight, before inoculating in LB broth. To culture the UPEC isolates in LB, colonies were chosen from the incubated agar plate and inoculated into 50 mL of fresh LB in 250 mL conical flasks and incubated at 37°C in a Brunswick G 25 shaker incubator (GMI, United States) at 180 rpm for 18 h.

### 2.2. Optimisation of D_2_O concentration

Following the procedure described in the growth conditions section, eight UPEC isolates were prepared and inoculated in fresh LB broth made with different concentration ratios of D_2_O/H_2_O ranging from 0 to 100% at intervals of 10%. The bacterial turbidity was adjusted to the standard optical density of 0.1 at 600 nm (OD_600nm_) and 200 μL of these cultures was transferred to a sterile 100-well plate (3 biological replicates), followed by monitoring the bacterial growth profiles at OD_600nm_ using a Bioscreen C spectrophotometer (Oy Growth Curves Ab Ltd., Finland). The following settings were used to run the instrument: preheating for 10 min, measurement intervals of 10 min, incubation temperature of 37°C, continuous medium shaking and 24 h of total experimentation.

### 2.3. Determination of trimethoprim minimum inhibitory concentration

In addition to *E. coli* MG1655, which was used as the standard strain for susceptibility testing to trimethoprim (TMP) with the minimal inhibitory concentration (MIC) value of 5 mg/L (Bhosle et al., 2020), all selected UPEC isolates were prepared following the procedure described in the growth conditions section. The prepared bacteria were then inoculated in fresh LB broth containing different concentrations of (5 mg/L, 7.5 mg/L and 10 mg/L), TMP and their growth profiles were recorded using the Bioscreen C, as described in the previous section.

### 2.4. Fourier transform infrared sample preparation and analysis

Following the incubation period and measurement of OD_600nm_, samples were centrifuged for 10 min at 5,000 × *g via* a benchtop Eppendorf centrifuge 5,910 R (Eppendorf Ltd., Cambridge, United Kingdom). The supernatant was removed and the biomass pellet was washed and resuspended 3 times using 1 mL of sterile physiological saline (0.9% NaCl) solution. The washed biomass was then stored at −80°C until further analysis. In order to adjust for variation in the collected biomass, all samples were normalized by adjusting the bacterial turbidity to the OD_600nm_ of 20. A total volume of 20 μL of prepared cell suspension was then spotted onto a cleaned 96-well FT-IR silicon plate and heated in a 55°C oven until visibly dry (~30 min). FT-IR spectra were then acquired in absorbance mode using an INVENIO infrared spectrophotometer equipped with an HTS-XT high throughput plate reader (Bruker Optics Ltd., Coventry, United Kingdom). Each sample was averaged over 64 spectral scans, with integration time of 1 s per scan, between the 4,000 and 400 cm^−1^ range with 4 cm^−1^ resolution (Winder et al., 2006). The extended multiplicative signal correction (EMSC) method was then used to scale all obtained spectra (Afseth and Kohler, 2012), and CO_2_ vibrations arising from the atmosphere (2,403–2,272 cm^−1^) were eliminated from the spectra and replaced with a linear trend (Alsberg et al., 1998), prior to performing any multivariate statistical analysis.

### 2.5. Optical photothermal infrared analysis

A series of serial dilutions from the bacterial solutions were prepared in sterilized Milli-Q water to achieve the appropriate cell number and allow for dispersed cell deposition and chemical imaging at the single-cell level. All bacterial suspensions were also visually assessed using a light microscope, to ensure the integrity of the cellular membrane is not compromised. A total volume of 10 μL of each concentration of samples was spotted on prewashed calcium fluoride (CaF_2_) slides, and air-dried in a desiccator. O-PTIR measurements were obtained from single bacterial cells on single-point mode, using a mIRage infrared microscope (Photothermal Spectroscopy Corp., Santa Barbara, United States). O-PTIR data (930–1,800 cm^−1^ and 1,970–2,320 cm^−1^, 2 cm^−1^ as spectral resolution, 10 scans per spectrum, with integration time of 10 s, and 3 spectra per bacterial cell) were collected in reflection mode through a Schwarzschild objective (40×, 0.78 NA); pump and probe beams consisting of a tunable QCL and a continuous-wave (CW) 532 nm laser, respectively (Lima et al., 2021, 2022). Single-frequency images were obtained by tuning the QCL to vibrational modes associated to amide I (1,655 cm^−1^) and C–D vibration (2,163 cm^−1^) at a 500 nm step size.

### 2.6. Data analysis

Once all the FT-IR and O-PTIR spectral data were collected, to remove spectra with low signal-to-noise ratio, all spectra with amide I signal intensities below 0.2 absorbance (arbitrary units) were excluded from further analysis. All data analyzes were carried out using MATLAB software version 2019a (MathWorks Inc., Natick, United States). The FT-IR and O-PTIR spectral data were then subjected to principal component analysis (PCA) to identify any natural clustering patterns. PCA is an unsupervised multivariate data analysis method, which is used to reduce the dimensionality of the data and as an exploratory data analysis tool. Its purpose is to extract important information from the data and to convert this information into a set of new orthogonal variables. PCA correspondingly signifies the pattern of similarity of the observations and the variables by demonstrating them as points in maps (Abdi and Williams, 2010). This was then followed by principal component-discriminant function analysis (PC-DFA; Manly and Alberto, 2016). As a supervised discriminant technique, DFA minimizes the variance within a group of samples from the same class and maximizes the variance between samples from different classes. Based on the true representation of the principal components (PCs) of the sample of interest, this technique calculates distances between the centers of each PC for clustering purposes (Goodacre et al., 1998).

## 3. Results

### 3.1. Bacterial growth profile

The growth profiles of all UPEC isolates in this study were monitored in LB broth medium with varying concentration ratios of D_2_O/H_2_O, to identify the maximum concentration of D_2_O to be used in the DIP experiment, without having any visible negative side effects on the growth behavior of the bacteria. Based on the results achieved, 80% D_2_O was chosen as the optimal concentration, as it displayed minimal side effects on the growth phenotypes of the *E. coli* UTI isolates and MG1655, while also allowing for the detection of a reliable C–D vibration upon the IR analysis of the samples at single-cell level (Supplementary Figures S1A,B).

Following the optimisation of D_2_O concentration, the concentration of 5 mg/L of TMP was identified as the minimum inhibitory concentration (MIC), which is in agreement with the standards for assessing antibiotic susceptibility reported in the literature (Bhosle et al., 2020). To ensure that bacterial growth was inhibited efficiently, the concentration of 10 mg/L (2 × MIC) was used in all the following experiments. According to the bacterial growth profiles (Supplementary Figures S1C,D), three susceptible (151, 122, and 147) and four resistant (6, 48, 123, and 143) isolates were identified; note that as expected *E. coli* MG1655 also showed no growth in the presence of TMP. Although at the initial first 6 h of the incubation period, a slight increase in OD_600nm_ of some of the susceptible isolates was observed, however, the final biomass yield remained almost unchanged. All resistant isolates showed similar behavior in terms of growth rate and final biomass yield under both TMP-treated and untreated (control) conditions (Supplementary Figure S1C and D).

### 3.2. Fourier transform infrared spectroscopy at the population level

To investigate the quantitative capability of FT-IR spectroscopy combined with the DIP strategy for detection of deuterium incorporation, the standard *E. coli* MG1655 strain was incubated in LB broth medium with various concentration ratios of D_2_O/H_2_O, from 0–100% with 10% intervals. Looking at the FT-IR spectra of these samples (Supplementary Figure S2A), the C–D vibrations in the 2,500–2,000 cm^−1^ region are clearly visible. These peaks appear due to the deuterium incorporation into bacterial cells, and hydrogen replacement, causing a change in the reduced mass (*μ*) (Muhamadali et al., 2015) of the corresponding vibrational bonds, resulting in the reduction of the naturally occurring C–H vibrational peaks (symmetric and asymmetric stretching vibrations of CH_3_ and CH_2_ functional groups) from the 3,000 to 2,800 cm^−1^ wavenumber region to the new occurrence of C–D peaks at 2,500–2,000 cm^−1^. Upon zooming into this region, a clear linear trend is visible, showing an increase in the absorbance of the four main C–D vibrations (2,471 cm^−1^, 2,418 cm^−1^, 2,195 cm^−1^, and 2,159 cm^−1^), corresponding directly to increasing D_2_O concentration in the medium from 0–100%. The PC-DFA scores plot of all the FT-IR spectral data, using the first 5 PCs accounting for 90.2% of the total explained variance (TEV), also displayed a clear D_2_O-dependent clustering pattern according to the DF1 axis, where samples with no D_2_O exposure were clustered on the far-left side of the axis, and samples with 100% D_2_O exposure were on the far positive side, and all other conditions were quantitatively clustered in between according to their D_2_O concentrations (Supplementary Figure S2B).

The loadings plot (Supplementary Figure S2C) showed the most significant vibrations that contributed to the observed clustering pattern, these include lipid bands (C–H and C–D) and protein bands (amide I and II), which allowed for the discrimination of bacterial groups grown in different concentrations of D_2_O. As it is demonstrated, the peaks at 2,925 cm^−1^ and 2,854 cm^−1^, assigned to C–H stretching vibrations (Stuart, 1997), are in the negative part of DF1, while the peaks at 2,471 cm^−1^ and 2,418 cm^−1^ are on the positive side. This suggested that, as expected with increasing D_2_O concentration, the C–H bands’ intensities decrease, while the C–D bands’ intensities increase.

**Figure 1:**
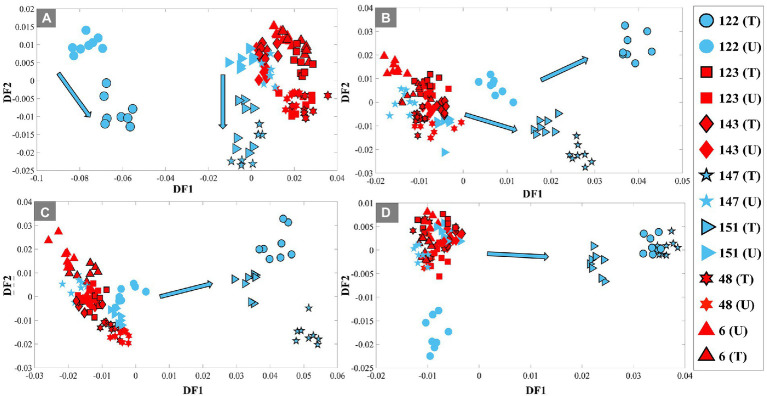
PC-DFA scores plots of pre-processed FT-IR spectral data (4,000–2,000 cm^−1^) of the UPEC isolates grown in LB broth containing 80% D_2_O, with (10 mg/l TMP) and without TMP at 4 different time points of 1 h **(A)**, 2 h **(B)**, 3 h **(C)** and 4 h **(D)**. TMP-susceptible isolates are shown in blue, while TMP-resistant isolates are shown in red. The black outline represents the TMP-treated (T) condition, while those without it represent the untreated (U) condition (control group). UPEC isolates investigated in this study are represented by different numbers and symbols. Blue arrows indicate the trend detected for the treated TMP-susceptible isolates.

Next, all selected UPEC isolates were incubated in LB broth medium containing 80% D_2_O, with and without exposure to TMP, and samples were collected at four different time periods (1, 2, 3, and 4 h) for FT-IR (bulk) and O-PTIR (single-cell) analysis. The pre-processed FT-IR spectral data of all isolates, in addition to common vibrational peaks of bacterial cells (Table 1), displayed four additional peaks (C–D signature peaks) at the 2,500–2,000 cm^−1^ wavenumber region, two of which, to our best of knowledge, are being reported for the first time using IR spectroscopy. As this study is aimed at using the C–D signatures for discriminating between TMP-sensitive and resistant isolates, in order to eliminate the contribution of the fingerprint region, subsequent analysis was mainly focused on the wavenumber region of vibrational peaks associated with deuterium incorporation (4,000–2,000 cm^−1^). Thus, all pre-processed FT-IR spectral data were combined and subjected to PC-DFA to identify any specific separation and clustering patterns resulting from the incorporation of deuterium. PC-DFA scores plots of the spectral data of all samples collected at 1 h timepoint at both conditions, TMP-treated and untreated, using 10 PCs accounting for 99.9% of the TEV (Figure 1A), showed slight discrimination between susceptible and resistant isolates according to DF1 axis. While both TMP-treated and untreated samples of the resistant isolates (123, 143, 48, and 6) displayed a tight cluster on the positive side of the DF2 axis, the TMP-treated samples of the sensitive isolates (122, 147, and 151) clustered on the negative side of DF2 axis, and the untreated samples were on the positive side. It is also perhaps worth noting that the DF1 axis is mainly dominated by the general biochemical differences between isolate 122 and the rest of the samples. Looking at the PC-DFA scores plots of the samples collected at subsequent timepoints (Figures 1B–D), it can be seen that the clustering patterns are starting to change from the 2 h timepoint onwards, while resistant isolates remain to form a tight cluster for both TMP-treated and untreated cells, the TMP-treated sensitive isolates seem to move away from the untreated samples and starting to separate according to DF1 axis. This separation is even more evident at the 3 and 4 h timepoints (Figures 1C,D), where the TMP-treated sensitive isolates are completely separated from the rest of the samples according to the DF1 axis. To explore other regions of the FT-IR spectral data, the full spectral range (4,000–400 cm^−1^) as well as the “fingerprint” region (2,000–400 cm^−1^) were also subjected to PC-DFA, following the same procedures mentioned above. Although the PC-DFA scores plots of these regions showed similar trend and clustering patterns, as expected, the degree of separation of TMP-treated groups of susceptible isolates from all other groups was not particularly evident until later timepoints (Supplementary Figures S3, S4).

**Table 1:** Common IR vibrational regions detected in bacteria, and their corresponding assignments (Stuart, 1997).

Wavenumber (cm^−1^)	Assignment	Nature of vibration
3,500–3,300	Proteins (Amide A)	N–H stretching
3,000–2,800	Lipids	C–H stretching
2,500–2,000	Lipids	C–D stretching
1,730	Lipids	C=O stretching
1,700–1,600	Proteins (Amide I)	C=O and C–N stretching
1,600–1,500	Proteins (Amide II)	N–H bending; C–N stretching
1,350–1,250	Proteins (Amide III)	C–N stretching; N–H bending
		C=O stretching; O=C–N; Other
1,200–900	Nucleic acids and polysaccharides	P=O, C=O and C–O–C stretching

**Figure 2:**
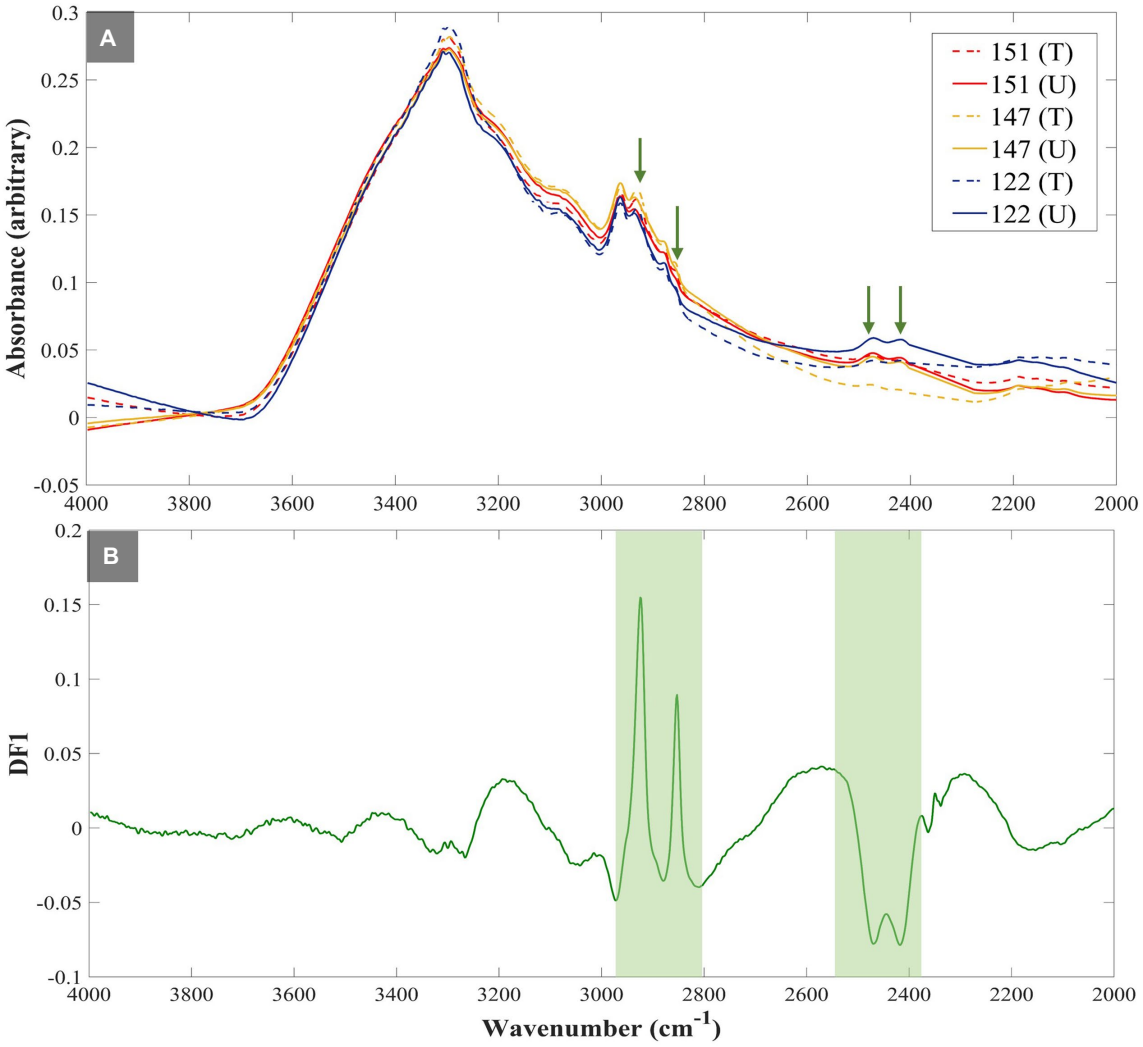
FT-IR spectra of susceptible UPEC isolates investigated in this study. The green arrows highlight the fatty acids’ CH_2_ (at 2,925 cm^−1^ and 2,854 cm^−1^) and C–D stretching vibrations (at 2,471 cm^−1^ and 2,418 cm^−1^) **(A)**, ‘DF1 loadings plot of FT-IR spectral data at 3h timepoint illustrating the most significant vibrational peaks (green strips) contributing to the discrimination patterns **(B)**. All spectra were normalized using extended multiplicative signal correction (EMSC). Each spectrum represents the average of four technical replicates. Different colored lines and numbers represent the different isolates, dotted lines represent treated groups, while untreated groups are represented as normal lines.

Figure 2A illustrates the averaged (*n* = 4) FT-IR spectra of susceptible UPEC isolates, which clearly highlights the difference in the intensities of C–D vibrations at 2,471 cm^−1^ and 2,418 cm^−1^ upon exposure of these isolates to TMP. This is of course as expected, as the susceptible isolates could not incorporate D_2_O when exposed to TMP, while the untreated cells were able to do so. Furthermore, the DF1 loadings plot (Figure 2B) of the FT-IR data at the 3 h timepoint (Figure 1C), also confirmed that the most substantial peaks contributing to the discrimination of TMP-treated susceptible isolates from all other samples include: fatty acid vibrations at 2,925 cm^−1^ and 2,854 cm^−1^ which belong to the asymmetric and symmetric stretching vibrations of CH_2_, respectively, and 2,471 cm^−1^ and 2,418 cm^−1^ which are associated with the C–D stretching vibrations. These findings further indicate that the bacterial strains assessed in this study were mostly distinguished based on their ability to incorporate deuterium, and consequently showed a decrease in the intensity of C–H vibrational bands, while the intensities of C–D bands were increased. However, as expected, such metabolic activity is hindered in susceptible strains upon TMP treatment, hence significantly lower deuterium incorporation is detected.

### 3.3. Rapid trimethoprim antibiotic susceptibility testing for Uropathogenic *Escherichia coli* isolates using optical photothermal infrared spectroscopy at the single-cell level

**Figure 3:**
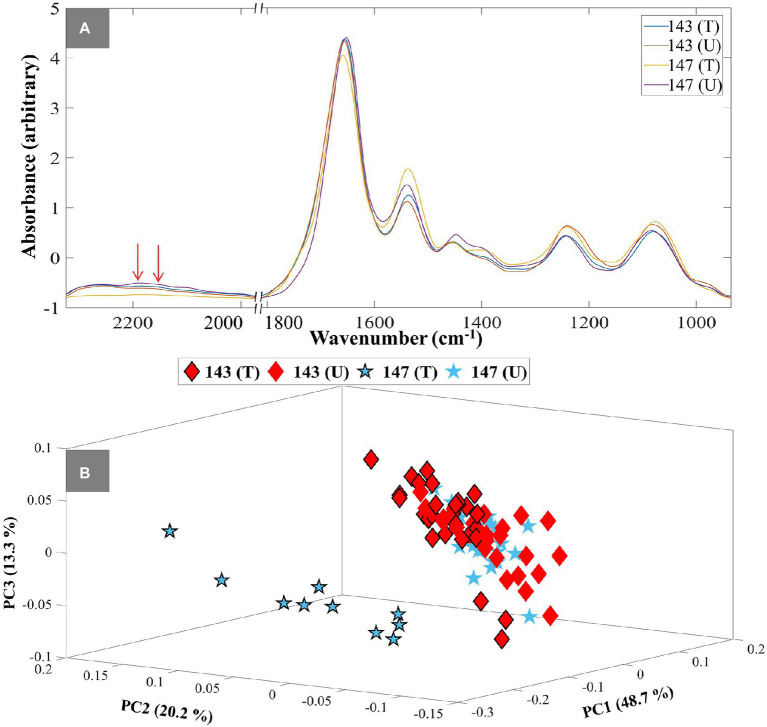
Single-cell O-PTIR spectral data of UPEC isolates at 3h timepoint **(A)**. Red arrows show the C–D stretching vibrational peaks at 2,159 cm^−1^ and 2,195 cm^−1^. Different colored lines and numbers represent the different isolates and are also indicated in the figure. The letters (T) and (U) indicate TMP-treated and untreated conditions. 3D PCA scores plot of O-PTIR spectral data at 3 h timepoint **(B)**. The blue colored stars represent susceptible isolates and the red colored diamonds represent resistant isolates. The black outline represents TMP-treated groups, while those without it represent untreated groups. Different symbols and numbers represent the different UPEC isolates in this study, the letters (T) and (U) indicate TMP-treated and untreated conditions. The percent total explained variance (TEV) is shown on the PC axes.

To demonstrate the application of the DIP strategy combined with IR spectroscopy at single-cell level, susceptible (isolate No. 147) and resistant isolates (isolate No. 143) were selected from the tested UPEC collection, and assessed by O-PTIR spectroscopy. Single-cell O-PTIR measurements were carried out on focused sections of 10 individual single bacterial cells from each condition, including TMP-treated and untreated, of susceptible and resistant isolates. The collected O-PTIR spectra of UPEC isolates at single-cell level (Figure 3A; Supplementary Figure S5) showed significant similarities to FT-IR spectra at bulk level. The results also evidently showed the difference in the intensities of C–D vibrations at 2,195 cm^−1^ and 2,159 cm^−1^ as a result of TMP-exposure. Following the data processing described above, the 3D PCA scores plot of the O-PTIR spectral data at the 3 h time point demonstrated a clear separation between the TMP-treated bacterial cells of the susceptible isolate from all other bacterial cells according to the PC1 axis with a TEV of 48.7% (Figure 3B). This is of course in agreement with the FT-IR findings (Figure 1), and as mentioned earlier it is due to the inability of the susceptible isolate (147) to incorporate D_2_O upon exposure to TMP, as they are not metabolically active, while other bacterial cells can continue to metabolise and incorporate deuterium. The 3D PCA scores plot of O-PTIR spectral data collected at the 4 h timepoint also illustrated similar trends, where TMP-treated susceptible cells clustered separately from all other bacterial cells, according to the PC1 axis with TEV of 53.5% (data not shown). However, O-PTIR spectral data of bacterial cells collected at 1 and 2 h timepoints did not display any distinct clustering for the susceptible and resistant isolates, as the C–D vibrations detected at these earlier timepoints were either below the detection limit of the O-PTIR instrument or unreliable, due to lower signal-to-noise ratio, at the single-cell level (data not shown). Hence, these data were not explored any further.

**Figure 4:**
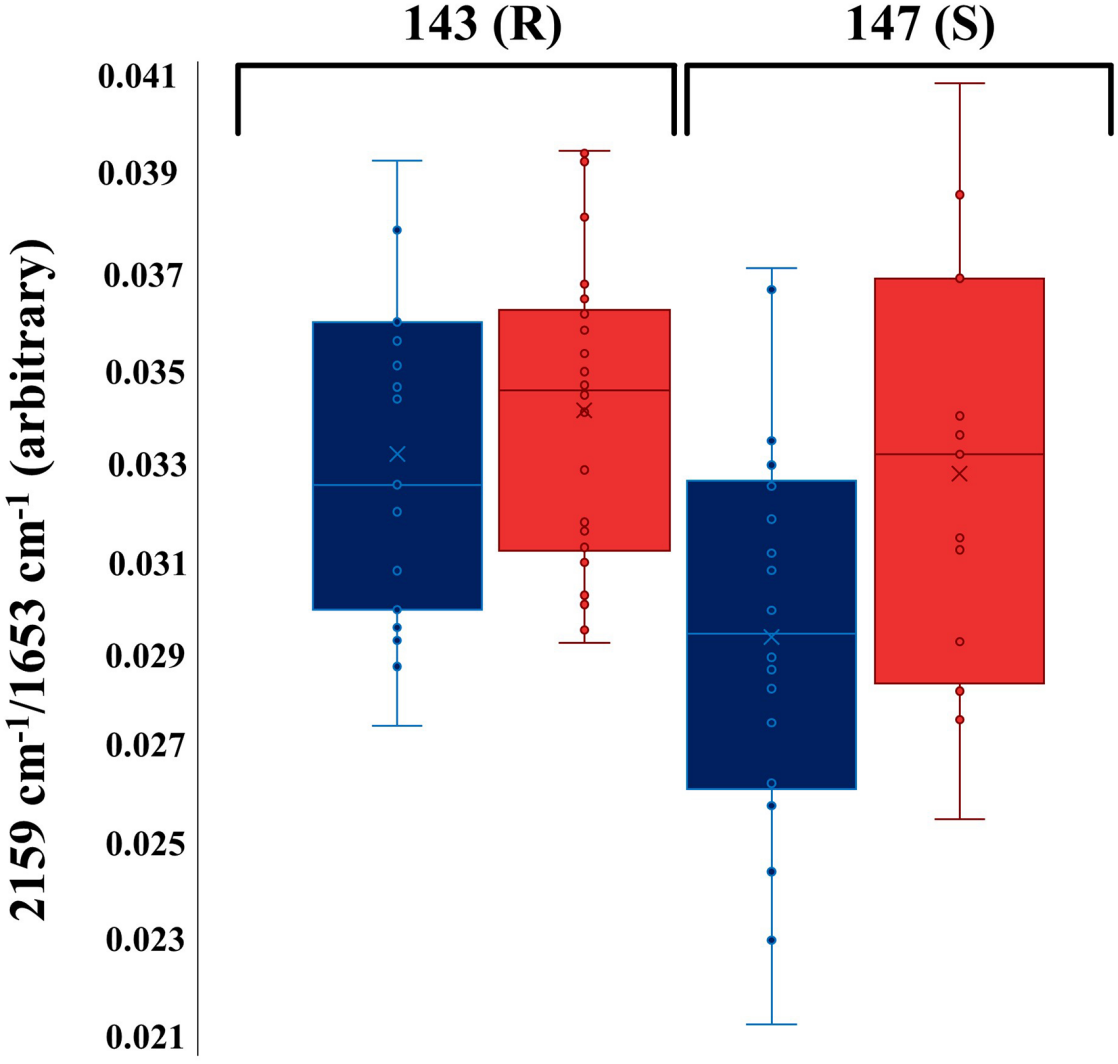
Box whisker plot of O-PTIR spectral data indicating the peak intensity ratio of the selected C–D stretching vibration (2,159 cm^−1^) to amide I (1,654 cm^−1^) at the 4 h timepoint. Blue and red box colors represent TMP-treated (T) and untreated (U) groups of bacterial cells, respectively. The numbers in the figure represent the UPEC isolates. (R) and (S) characterize the resistant and susceptible strains, respectively.

These findings highlight the sensitivity and reproducibility of O-PTIR for discrimination of susceptible and resistant bacterial strains. In order to explore the potential application of using only the C–D vibration for discrimination of resistant and sensitive isolates to TMP, the peak intensity of the C–D vibration at 2,159 cm^−1^ was normalized against the well-known amide I vibration at 1,654 cm^−1^, and compared as box whisker plot (Figure 4). The results indicated that the TMP-treated bacterial cells of the susceptible isolate (147) had incorporated less deuterium in comparison to the untreated bacterial cells from the same isolate (Figure 4). While according to the peak intensity ratio of the selected C–D band to amide I, the resistant isolate (143) incorporated almost the same amount of deuterium under both culturing conditions (Figure 4).

**Figure 5:**
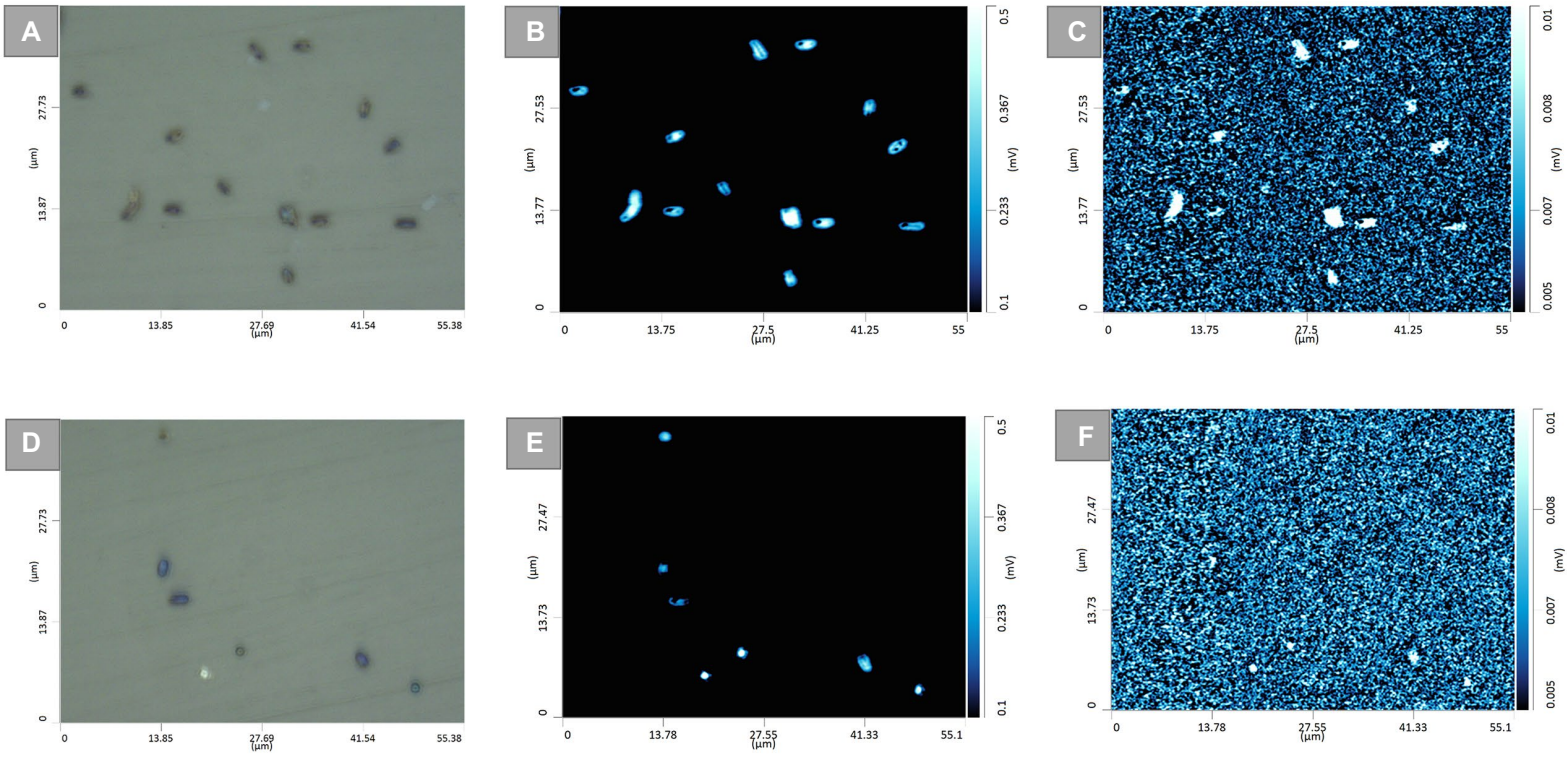
Optical images of untreated **(A)**, and TMP-treated **(D)** *E. coli* cells (isolate 147, TMP-sensitive). The corresponding single-frequency O-PTIR images were obtained using the amide I vibration at 1,655 cm^−1^ for the untreated **(B),** and TMP-treated **(E)** cells, and the C–D vibration at 2,163 cm^−1^ for the untreated **(C)**, and TMP-treated **(F)** cells.

To demonstrate further the application of such a strategy for differentiating TMP-sensitive from resistant isolates at single-cell level, O-PTIR single-frequency images of the two *E. coli* isolates, TMP-susceptible (Figure 5) and resistant (Supplementary Figure S6), grown under both untreated and TMP-treated conditions were collected and compared by targeting the amide I and the C–D vibrational bands. Optical images of untreated (Figure 5A) and TMP-treated (Figure 5D) *E. coli* cells (isolate 147, TMP-susceptible) highlighted the position of individual cells. These bacterial cells grown under both untreated (Figure 5B) and TMP-treated (Figure 5E) conditions also displayed significant contrast from the background using the 1,655 cm^−1^ peak assigned to the amide I band of proteins. However, under the same field of view using the peak at 2,163 cm^−1^, assigned to C–D vibration, while the untreated TMP-susceptible *E. coli* cells (Figure 5C) were clearly visible, suggesting that they are metabolically active and incorporating deuterium, the TMP-treated cells were hardly detected, suggesting that they are not metabolically active and therefore, they could not incorporate deuterium (Figure 5F). This is while, both untreated (Supplementary Figure S6B) and treated (Supplementary Figure S6D) TMP-resistant *E. coli* cells (isolate 143) were clearly detected in the single-frequency images using both peaks at 1,655 cm^−1^ and 2,163 cm^−1^, confirming the deuterium incorporation under both growth conditions.

## 4. Discussion

There is no doubt that AMR has become one of the most pressing public health problems in the 21st century, threatening lives with a growing range of bacterial infections which in most cases no longer respond to commonly available medications and antibiotics (Prestinaci et al., 2015). Rapid recognition of antimicrobial-resistant bacteria accelerates the initiation of appropriate and targeted treatment and effective antibiotic therapy preventing further development and spread of AMR (Lery, 2002; Ellis et al., 2007; Marshall and Levy, 2011). During the past few decades, stable-isotope labeling has been widely used to study microbial ecophysiology (Hernández et al., 2017), biogeochemical cycles (Radajewski et al., 2000), biotechnological procedures (Coyotzi et al., 2016) and human-disease interactions (Egert et al., 2007; Xu et al., 2021). This is because vibrational spectroscopic techniques (including infrared and Raman spectroscopies) have been employed for probing the cellular uptake of these stable isotopes and consequently detect chemical vibrations arising from the selective incorporation of isotopically labeled molecules (Haris et al., 1992; Berry and Loy, 2018). In spite of the extensive use of IR spectroscopy for AMR identification (Salman et al., 2017; Sharaha et al., 2017), the diffraction limit of the IR light has been the main barrier for acquiring IR chemical fingerprints at the submicron spatial resolution, especially when it comes to investigating individual bacterial cells (typically 1–2 μm). For this reason, research studies using IR spectroscopy were mostly conducted at the population level. This is while, in contrast to IR spectroscopy, Raman spectroscopy has proven to be an efficient fingerprinting tool for investigating samples at the single-cell level, as the diffraction limit provides an appropriate spatial resolution to study single cells (Bechtel et al., 2014). To overcome the diffraction limitations, by employing continuous-wave visible lasers (532 nm), O-PTIR measures the photothermal effect induced in IR-active samples by irradiating them with a tunable IR laser (Kansiz et al., 2020). Therefore, in this study, we aimed to explore the application of the DIP strategy combined with FT-IR and O-PTIR spectroscopy for the detection and discrimination of AMR in UPEC isolates at both population and single-cell levels, respectively. The results of the FT-IR spectral data revealed that the corresponding intensities of a group of particular bands (C–D signature peaks; Supplementary Figure S2A) can be used for the discrimination of the susceptible and resistant bacteria to TMP. The PC-DFA scores plots of the spectral data collected at 1, 2, 3, and 4 h timepoint (Figures 1A–D) indicated that the discrimination is not strain-dependent, and is instead due to the bacterial response to TMP treatment and its effect on the ability of the cells to undergo normal metabolism and thereby incorporate deuterium, which causes C–H vibrational bands that are generally assigned to fatty acids (but can of course be from any chemical that contains the C-H functional group) being shifted to the silent region (2,800–1,800 cm^−1^; Naumann, 2000). The PC-DFA loadings plot (Figure 2B), also confirmed these findings as it displayed an increase in C–D vibration, upon incorporation of deuterium, while showing a decrease in the CH_2_ vibrations, suggesting that the investigated bacterial cells are metabolically active, meaning that the selected antibiotic does not affect the growth of those isolates, in other words the isolate is resistant to the selected antibiotic. It has been reported that lipids of both heterotrophic and autotrophic organisms contain a high proportion of water-derived protons (or D^+^ in the presence of D_2_O; Sessions et al., 2002; Valentine et al., 2004; Campbell et al., 2009; Zhang et al., 2009; Wegener et al., 2012) as during the reduction of NADP and NADH, H^+^/D^+^ from H_2_O/D_2_O are transferred to these electron carries that are associated with cellular metabolic activity. H^+^/D^+^ are then incorporated into lipids through fatty acid biosynthetic pathways forming C–D bonds (Sessions et al., 2002; Valentine et al., 2004; Zhang et al., 2009). In addition to lipids, D can also be incorporated into amino acids and carbohydrates through other biosynthetic pathways (Fischer et al., 2013; Justice et al., 2014). The results of the single-cell O-PTIR spectral data (Figure 3A; Supplementary Figure S5) displayed a significant resemblance to FT-IR showing efficacy of O-PTIR for identification of AMR. The PCA scores plot of the O-PTIR spectral data at the 3 h timepoint (Figure 3B) successfully discriminated the TMP-treated bacterial cells of the susceptible isolate from all other bacterial cells. Also, the intensity ratio of the C–D stretching vibration at 2,159 cm^−1^ to amide I (Figure 4) in TMP-treated bacterial cells of the susceptible isolate was less than untreated bacterial cells of the susceptible isolate. This is while, this ratio was almost equal in both treated and untreated bacterial cells of the resistant isolate, emphasizing that these bacteria are resistant to the selected antibiotic, remain metabolically active and keep incorporating deuterium, highlighting the application of DIP strategy combined with IR spectroscopy for identification of AMR at single-cell level. In addition, in line with the single-cell O-PTIR spectral data, the O-PTIR single-frequency (2,163 cm^−1^, assigned to C–D vibration) images of the TMP-susceptible bacterial cells grown in the presence of trimethoprim (Figure 5F), clearly displayed the significantly reduced ability of these cells to incorporate deuterium, compared to the cells grown under the untreated conditions (Figure 5C). This is while, the O-PTIR single-frequency images of the TMP-resistant bacterial cells’ under both TMP-treated and untreated conditions (Supplementary Figures S6C,F) demonstrated distinct resemblance. It is also perhaps worth noting that some of the differences detected within the same bacterial community (Figure 4) could also be attributed to the high special resolution provided by the O-PTIR technology, which allows for the detection of such naturally occurring heterogeneity in the metabolic activity of the community at the single-cell level.

## 5. Conclusion and outlook

In this study for the first time, we report the employment of O-PTIR combined with deuterium isotope probing and multivariate statistical analysis, as an efficient tool for the AST of UPEC isolates at the single-cell level. The use of D_2_O is particularly powerful as water is involved in many metabolic reactions and so the incorporation of deuterium into the cell will occur relatively quicker, while also being more cost effective, compared to the use of other stable isotopes such as ^13^C or ^15^N. According to our findings, the collected infrared spectra at bulk and single-cell levels showed substantial resemblances and reproducibility in the clustering pattern and were in complete agreement. FT-IR spectroscopy was also employed to demonstrate four C–D signature peaks (2,471 cm^−1^, 2,418 cm^−1^, 2,195 cm^−1^, and 2,159 cm^−1^), of which, to our best of knowledge, two of them (2,471 cm^−1^ and 2,418 cm^−1^) are being reported here for the first time. However, unfortunately, these two vibrations were not detected at the single-cell level, as the spectral range of the O-PTIR instrument was limited to a specific range (2,320–1,970 cm^−1^ and 1,800–930 cm^−1^), due to the restrictions in the maximum number of QCLs that can be used at any one time. To overcome this limitation, future studies can employ a tunable OPO-pulsed infrared laser to cover the higher wavenumber region of infrared radiation (Spadea et al., 2021). O-PTIR spectroscopy, individually or when combined with Raman spectroscopy such as the study conducted by Lima et al. (2022), can also be a powerful fingerprinting tool allowing scientists to gain novel insight into biological systems and bioprocesses of single bacterial cells. The results reported in this study successfully demonstrated that O-PTIR spectroscopy can be employed as a promising tool for identifying AMR at the single-cell level.

## Data availability statement

The raw data supporting the conclusions of this article will be made available by the authors, without undue reservation.

## Author contributions

SS: experimental design, sample collection and preparation, FT-IR and O-PTIR data analysis, data interpretation, and manuscript writing. CL: sample preparation, O-PTIR data analysis and manuscript preparation. YX and SA: data analysis and interpretation. RG: experimental design, data interpretation, and manuscript preparation. HM: principal investigator, experimental design, sample collection and preparation, FT-IR and data analysis, data interpretation, and manuscript preparation. All authors contributed to the article and approved the submitted version.

## Conflict of interest

The authors declare that the research was conducted in the absence of any commercial or financial relationships that could be construed as a potential conflict of interest.

## Publisher’s note

All claims expressed in this article are solely those of the authors and do not necessarily represent those of their affiliated organizations, or those of the publisher, the editors and the reviewers. Any product that may be evaluated in this article, or claim that may be made by its manufacturer, is not guaranteed or endorsed by the publisher.
