# Supplementary material for: Optical photothermal infrared spectroscopy: A novel solution for rapid identification of antimicrobial resistance at the single-cell level via deuterium isotope labeling
Source: Front Microbiol. 2023 Feb 1;14:1077106. doi: 10.3389/fmicb.2023.1077106 (PMC9929359; doi:10.3389/fmicb.2023.1077106)
Supplement: Supplementary file 1 [file Data_Sheet_1.pdf]

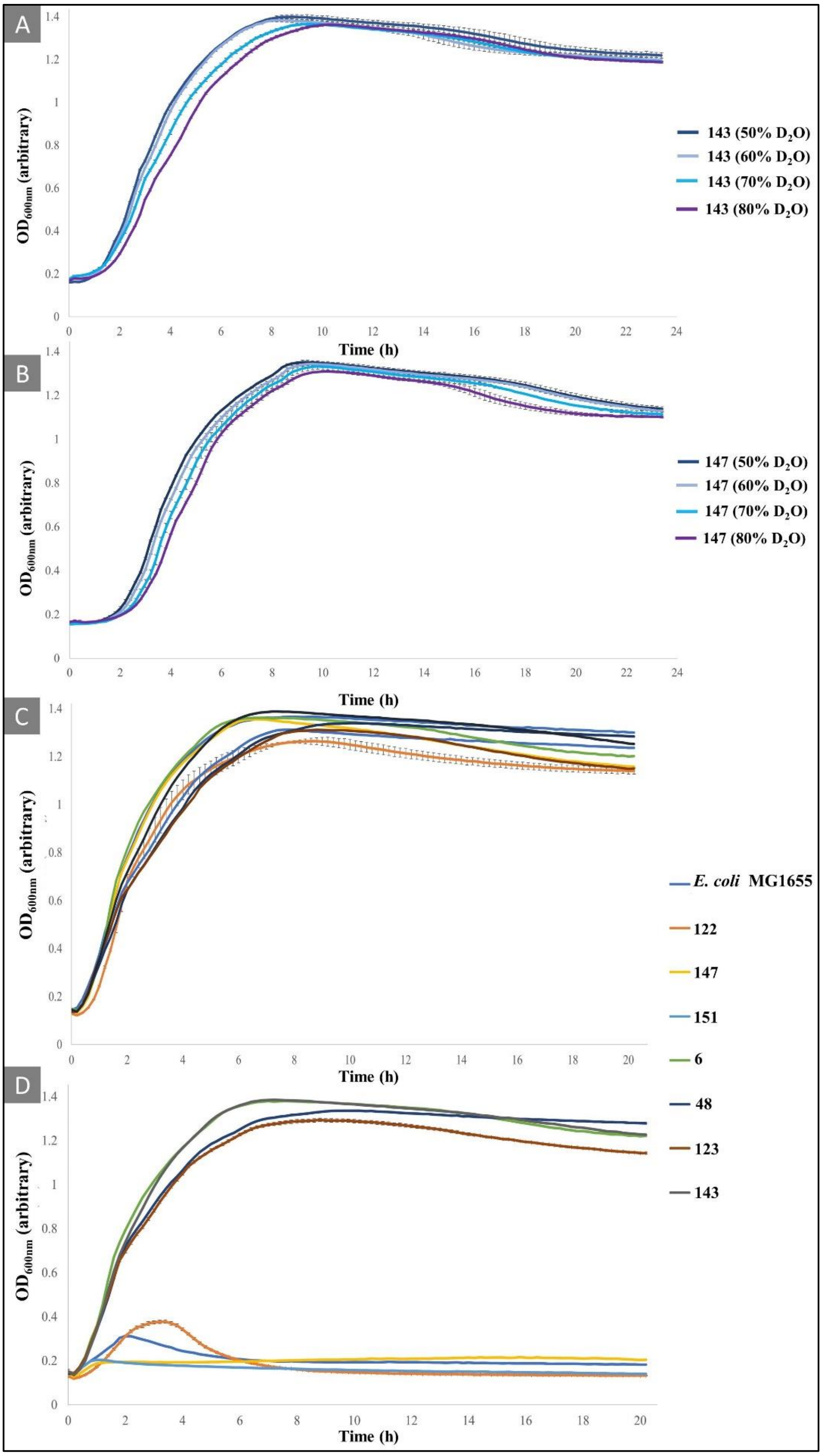

Figure S1| Growth profiles of selected UPEC isolates of 143 (**A**) and 147 (**B**) in LB containing different concentrations of D_2O (50%, 60%, 70% and 80%). Growth profiles of selected UPEC isolates in LB (**C**) and LB containing 10 mg/L TMP (**D**) for determination of MIC. The growth curves are presented as the average of three biological replicates. Due to the high reproducibility of the data and to improve the visibility of the individual growth curves, the standard deviation of a subset of the isolates is plotted as representative. Different numbers and coloured lines represent the different isolates and are also indicated in the figure.

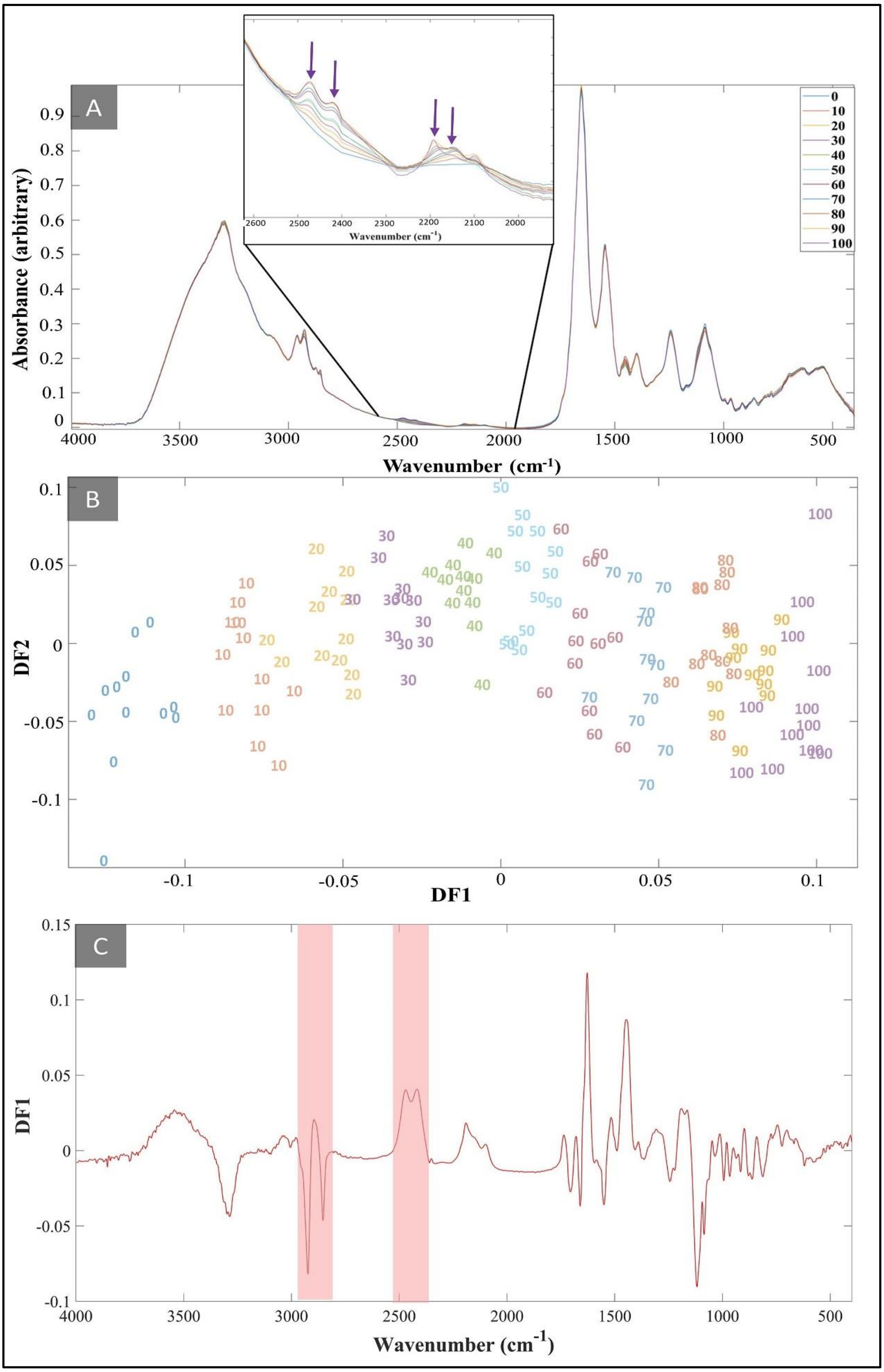

Figure S2| The FTIR spectra of *E. coli* MG1655 grown in different concentrations of D_2O **(A)**. The purple arrows highlight the main C–D vibrational peaks. The legend highlights different concentrations of D_2O (in %) used in the culture medium. PC-DFA scores plot of FTIR spectral data of *E. coli* MG1655 grown in different concentrations of D_2O **(B)**. The coloured numbers illustrate the percentages of D_2O used in the growth medium. DF1 loadings plot showing the important peaks contributing to clustering patterns of

bacterial groups grown in different concentrations of D_2O **(C)**. The red strips highlight the wavenumber position of C-H (2923^{-1} and 2854 cm $^{-1}$) and C–D peaks (2467cm^{-1} and 2414 cm^{-1})

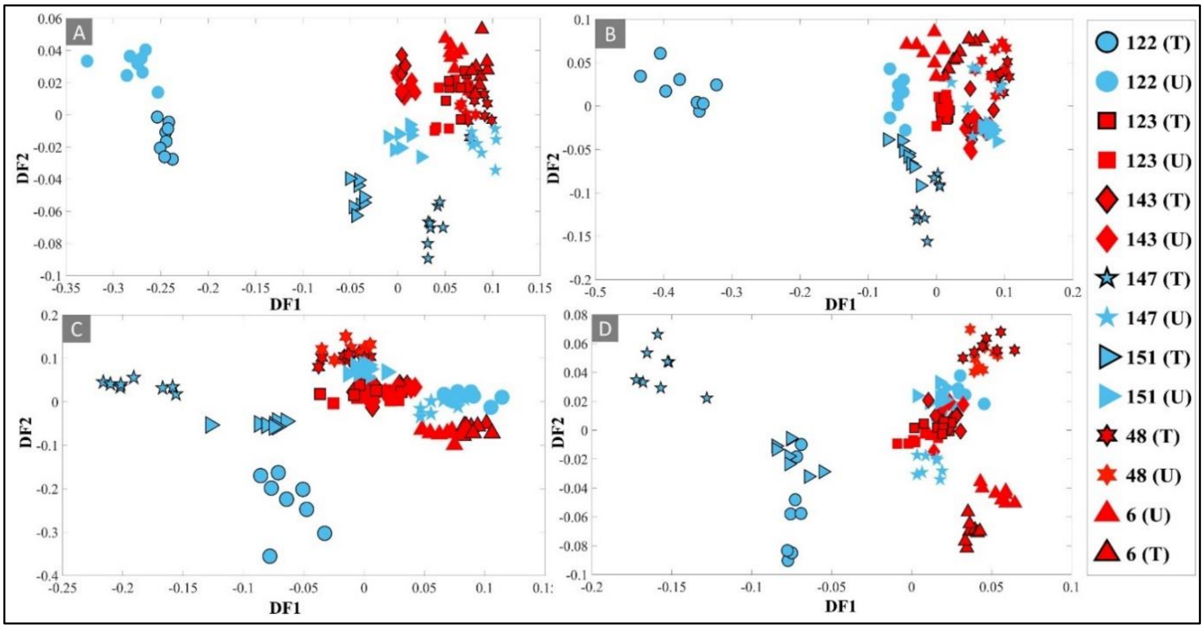

Figure S3|PC-DFA scores plots (using 10 PCs) of pre-processed FTIR spectral data (4,000-400 cm^{-1}) of the UPEC isolates grown in LB containing 80% D_2O and 10 mg/L TMP at 4 different time points of, 1 h (**A)** (TEV= 99.44%), 2 h **(B)** (TEV = 99.35%), 3 h **(C)** (TEV = 99.15%), and 4 h **(D)** (TEV = 99.42%). The blue colour represents susceptible isolates and the red colour represents resistance isolates to TMP. The black outline represents treated (T) groups of isolates, while those without it represent untreated (U) groups of isolates. Different symbols represent the different UPEC isolates in this study, and the coding for this is provided in the figure.

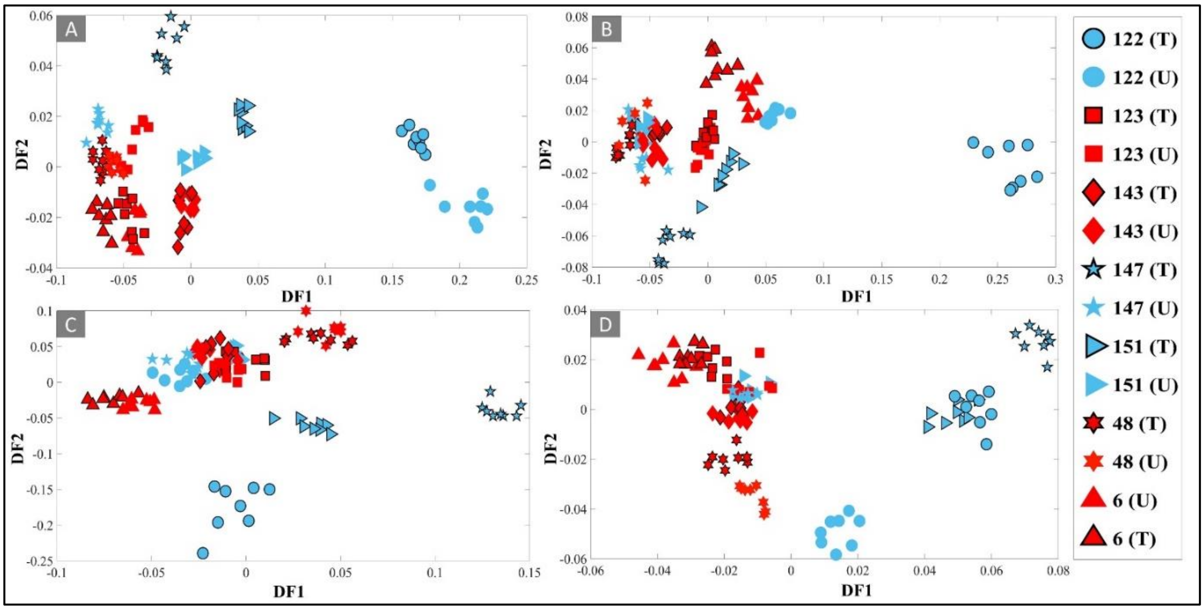

Figure S4|PC-DFA scores plots (using 10 PCs) of pre-processed FTIR spectral data (2,000-400 cm^{-1}) of selected UPEC isolates grown in LB containing 80% D_2O and 10 mg/L TMP at 4 different time points of 1 h **(A)** (TEV= 99.09%), 2 h **(B)** (TEV= 98.61%), 3 h **(C)** (TEV= 98.63%), and 4 h **(D)** (TEV= 98.55%). The blue colour represents susceptible isolates and the red colour represents resistance isolates to TMP. The

black outline represents treated (T) groups of isolates, while those without it represent untreated (U) groups of isolates. Different symbols represent the different UPEC isolates in this study, and the coding for this is provided in the figure.

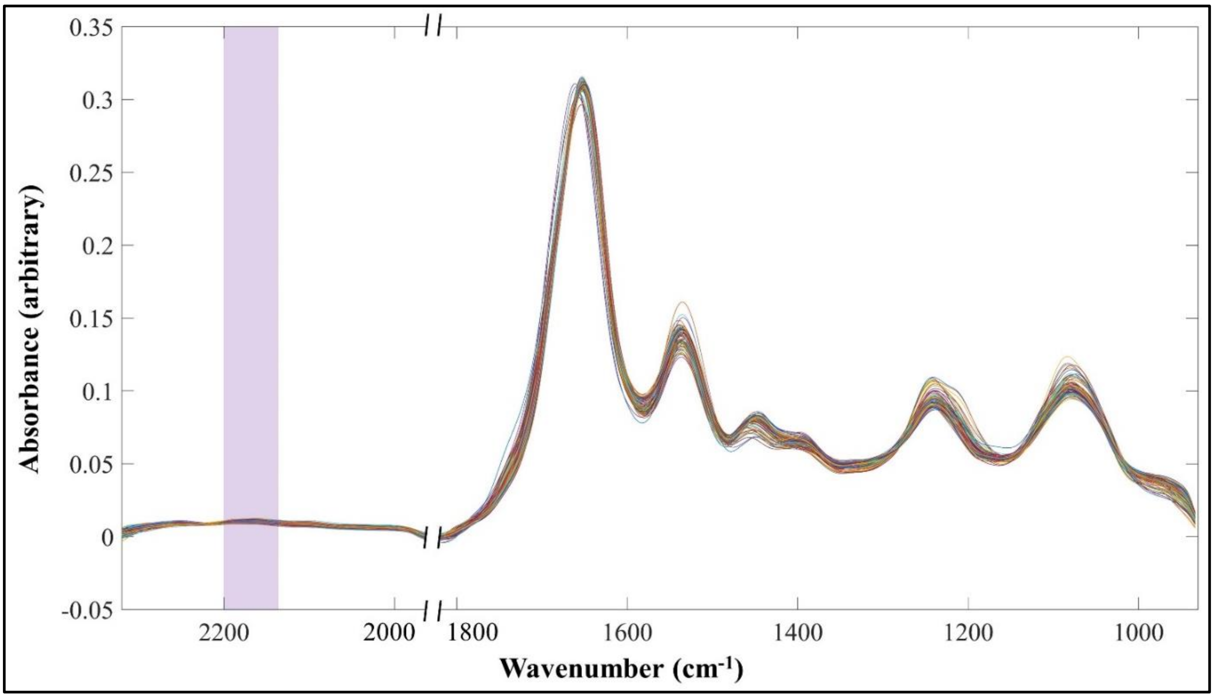

Figure S5| Single-cell O-PTIR spectra of UPEC isolates in 3 h timepoint. All spectra were normalised using extended multiplicative signal correction (EMSC). Different coloured lines represent the investigated UPEC isolates (isolate No. 147 and 143). The purple strip highlights the C–D vibrational peaks region used for the discrimination of susceptible and resistant isolates.

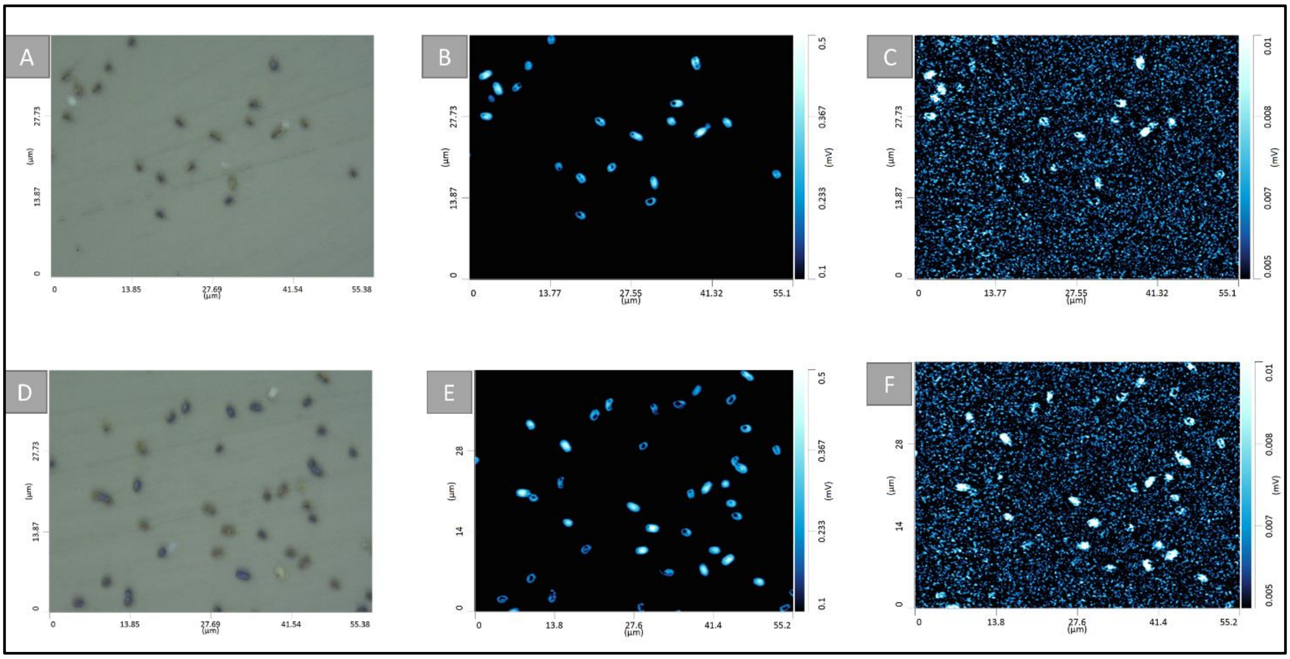

Figure S6| Optical images of untreated (A), and TMP-treated (D) *E. coli* cells (isolate 143). The corresponding single-frequency O-PTIR images were obtained using the amide I vibration at 1655 cm^{-1} for

the untreated (B), and TMP-treated (E) cells, and the C–D vibration at 2163 cm^{-1} for the untreated (C), and TMP-treated (F) cells.
